# Ameliorative Effects of *Thunbergia erecta* L. Leaves Against the Initiation of Hepatocarcinogenesis Induced by Diethylnitrosamine in the Rat Model

**DOI:** 10.1007/s12010-022-04292-x

**Published:** 2023-01-28

**Authors:** Fatma Sayed Abdel-Aal Farag, Hend Mohamed Anwar, Tarek Aboushousha, Hala Sh. Mohammed, Lotfi Diab Mousa Ismail

**Affiliations:** 1 Pharmacognosy and Medicinal Plants Department, Faculty of Pharmacy (Girls), Al-Azhar University, Cairo, 11651 Egypt; 2 Department of Biochemistry, National Organization for Drug Control & Research, Cairo, Egypt; 3 Department of Pathology, Theodor Bilharz Research Institute, Kornaish El-Nile, Warrak El-Hadar, P.O. 30 Imbaba, Giza, 12411 Egypt; 4 Pharmacognosy and Medicinal Plants Department, Faculty of Pharmacy (Boys), Al-Azhar University, Cairo, 11651 Egypt

**Keywords:** Thunbergia, Apoptosis, Caspase, Acacetin, Phenolic, Diethylnitrosamine

## Abstract

*Thunbergia erecta L*. contains cytotoxic and liver-protective compounds. *Thunbergia erecta* L. leaves were macerated in 70% aqueous ethanol, then fractionated with ethyl acetate (9.3 g) and butanol (12.7 g), and attenuated Den-induced liver cancer in a Wistar rat experimental model. Ethyl acetate and butanol fractions were chromatographed using column chromatography and solid-phase extraction (SPE); Vicenin-II (1), kaempferol (2), biochanin A, sissotrin 7-*O*-β-glucopyranoside (3), gentianose (4), acacetin 7-*O*-β-glucopyranoside (5), apigenin 7-*O*-β-glucopyranoside (6), and rosmarinic acid (7) were extracted, and their structures were determined using NMR spectroscopy and ESI-mass spectrometry. Sixty rats were divided into six groups (ten each): control group, Den group, doxorubicin/Den-treated group, butanol fraction/Den-treated group, and isolated acacetin 7-*O-*β*-*glucopyranoside/Den-treated group. The liver enzymes and proinflammatory biomarkers were used to estimate the liver function. In addition, liver tissues were collected for analysis of oxidative stress markers, gene expression, and histopathology. There is a significant increase in the levels of liver enzymes, AFP, and TNF-ἁ. This was conveyed by a significant increase of IL-1 and caspase-3, elevation of MDA and reduction of GSH, and suppression of Bcl2 and elevation of Bax expression. All parameters in butanol, ethyl acetate fractions, and isolated acacetin 7-*O*-β-glucopyranoside (major constituents) of *T. erecta* L. were significantly improved to values close to those of the control group.

**Supplementary Information:**

The online version contains supplementary material available at 10.1007/s12010-022-04292-x.

## Introduction

Plant-derived natural compounds have played a vital role in human life. Many scientific types of research have revealed that medicinal plants may be used as natural remedies and are now being used as an alternative for medications [1. ]. *Thunbergia erecta* L. is vigorous, a woody shrub which belongs to Acanthaceae family. This shrub has small, ovate leaves with entire margins borne opposite on thin, brown stems [2. ]. The purple flowers have a yellow throat and may appear singly or in small clusters. This plant produces rounded seed capsules that end in a beak. *Thunbergia* genus has ornamental value and is native to tropical regions of Africa and South Asia [3. ]. *Thunbergia* species have a number of bioactive chemicals with established pharmacological effects include alkaloids, naphthalene, coumaroyl malic acid, benzyl β-glucopyranoside, delphinidin, apigenin, and phenolic compounds such as tannin, feruloyl malic acid, and rosmarinic acid [4. , 5. ]. Traditionally, *Thunbergia* species leaves, stems, and roots was used as anti-inflammatory and antipyretics agents [6. ]. It has been also reported to possess antibacterial activities against gram positive as well as gram-negative bacteria such as *Escherichia coli*, *Klebsiella pneumonia*, and *Streptococcus pyogenes* [7. ]. *Thunbergia* species also exhibit antinociceptive and antitumor [8. ], cytotoxic, and antioxidant activities [9. , 10. ]. Regarding the previous reports and our phytochemical analysis, it has been revealed that *T. erecta* L. contains several bioactive compounds which have ameliorative effects against hepatocellular carcinoma. Liver cancer is one of the world’s most prevalent diseases, especially in Asia and Africa [11. ]. Hepatocellular carcinoma (HCC), the fourth most common cause of cancer mortality, accounts for 90% of all liver cancer [12. , 13. ]. Diethylnitrosamine (Den) is a well-known hepatocarcinogenic agent found in cigarette smoke, water, cured and fried foods, cheese from cheddars, chemicals from the farming industry, cosmetics, and pharmaceutical products [14. , 15. ]. Despite continuous advances in managing chronic liver diseases, tumor detection, and treatment, the prognosis of HCC remains lower in comparison to other tumors [16. ]. The incidence of high mortality and associated side effects following chemotherapy and/or radiotherapy increase the demand for alternative medicines for cancer treatment. Not surprisingly, many potent anticancer compounds have been isolated from plants, e.g., doxorubicin, taxol, etoposide, cisplatin, vinblastine, vincristine, and topotecan [17. ]. This has led to the current interest in alternative medicine that will aid in therapy and help to avoid its unjustifiable use. Liver enzymes AST (aspartate transaminase), ALT (alanine transaminase), and ALP (alkaline phosphatase protein) are indicative of diagnosis and severity of the liver injury [18. ] and when hepatocytes were injured or destructed the pro-inflammatory factors like TNF-α, IL-1β, IL-6, and ROS (reactive oxygen species) are released [19. ], MDA (malonaldehyde) is an indicator of lipid peroxidation [20. ]. Caspases are a family of protease enzymes playing essential roles in programmed cell death [21. ]; α-fetoprotein (AFP) is an important tumor marker with the best sensitivity and specificity for hepatocellular carcinoma (HCC) diagnosis and monitoring of its therapy [22. ]. The members of the Bcl-2 family involving of both proapoptotic and anti-apoptotic proteins contribute to the regulation of programmed cell death in hepatocytes [23. , 24. ]. Numerous of the genetic amendments of fibrosis initiate an imbalance in the proapoptotic and anti-apoptotic proteins of the Bcl-2 family [25. ]. The anti-apoptotic proteins, including Bcl-2, is known to hinder mitochondrial apoptotic pathway by delaying the release and oligomerization of proapoptotic proteins, and is downregulated in fibrotic cell [26. ]. On the other hand, proapoptotic members of the Bcl-2 family, such as Bax, activate mitochondrial apoptosis by enabling pore development and cytochrome *c* release from the inner mitochondrial membrane with consequent initiation of caspases resulting in cell death. These proapoptotic members are over expressed in fibrosis [27. ]. An amendment in Bax/Bcl-2 ratio plays a critical role in determining whether a cell should switch towards proliferation or apoptosis [28. ]. Den was therefore used in this study to construct a liver cancer model in Wistar rats. The potential bioactive chemical components of *T. erecta* L. leaf extract and their anti-cancer effects were then studied.

## Materials and Methods

### Plant Material

 *T. erecta *L. fresh leaves were harvested in May 2018 from the Attia Shoala flower plantation in Kaliobeya, Egypt. Mrs. Therese Labib, botanical specialist, Department of Flora and Taxonomy, El-Orman Garden, Giza, Egypt, generously identified the plant. A voucher specimen of the plant (Reg. No. T7) was deposited at the Herbarium of the Pharmacognosy and Medicinal Plants Department at Al-Azhar University’s Faculty of Pharmacy in Cairo, Egypt.

### Instruments

^1^H and ^13^CNMR (king Abdul-Aziz University, Jeddah, Saudi Arabia, BRUKER 850 MHz for ^1^H and 213 MHz for ^13^C, Cairo University-Faculty of Pharmacy BRUKER 400 for ^1^H and 100 MHz for ^13^C), Glass-Col motor-driven homogenizer (USA), UV/Visible Spectrophotometer (Unicam )5625 UV/VIS Spectrometer USA), and XEVO TQD triple quadruple ESI-MS (MA01757 USA, mass spectrometer).

### Chemicals and Reagents

Were purchased from Sigma Chemical Company, St. Louis, MO, USA. Colorimetric ELISA kit (serum AFP Abia Ref. DK.045.01.3, Rat αTNF Sino Gene Clon Ref. SG20127), Bio-diagnostics kit (AL 1031kit for serum ALT, AS 1061kit for serum AST, Gpl kit for serum ALP), IL-1beta and Caspase-3 bio kits, SMART Scribe™ Reverse Transcriptase (Clontech Laboratories, Inc. A Takara Bio Company), TRIzol® reagent (Invitrogen, Sigma-Aldrich, St. Louis, MO), and SYBR® Green PCR Master Mix (QIAGEN) for gene expression PCR. All other solvents and reagents were of the highest grade commercially available.

### Extraction, Fractionation, and Purification

Air-dried leaves powder (1.4 kg) of *T. erecta* L. macerated using 70% aqueous ethanol (5 × 3 L) at 25 ± 2 °C. The extract was filtered, and the filtrate was evaporated using a rotary evaporator (Buchi Co., Switzerland) at 70 °C. The total leaf extract (155 g), 0.1 g of leaf extract used for quantitative estimation of total flavonoid and phenolic compounds, and the remaining extract fractionated at 25 ± 2 °C ethyl acetate (3 × 1 L) and butanol (5 × 1 L), yielding 9.3 g, and 12.7 g respectively. The ethyl acetate fraction was applied on a successive silica gel column (100 g, 70 × 4 cm) using CH_2_Cl_2_:CH3OH to obtain compounds **1**, **2**, and **3** while the butanol fraction was subjected to polyamide S6 CC (100 g, 80 × 5 cm) eluted with H2O/EtOH mixtures up to EtOH. By using PC, UV light, and spray reagents, similar fractions were collected to obtain nine collective fractions (I–IX), which were purified by applying on successive silica gel columns using EtOH and CH_2_CL_2_. Then, the sub-fractions were purified using sephadex LH-20/ethanol, which obtained compounds **4**, **5**, **6**, and **7**.

### Total Phenolic and Flavonoid Assessment

Total phenolic content was evaluated using the Folin-Ciocalteau assay technique [23. ], and total flavonoids were quantified using [29. ].

### Biological Study Methods

#### Animals

Adult male Wister albino rats weighing between 100 and 120 g were obtained from the National Organization for Drug Control and Research (NODCAR), Cairo, Egypt, were used. Rats were housed in standard polypropylene cages in the institute’s animal house facility at 25 °C with 50–60% relative humidity, 12-h light-dark cycles, and free access to food and water. Care and use of the animals were conducted under the supervision of the Animal Ethics Committee of the National Organization for Drug Control and Research (NODCAR) with an approved ethical number (NODCAR/II/13/2020). The study was also conducted according to the National Regulations on Animal Welfare and the Institutional Animal Ethical Committee (IAEC).

#### Experimental Protocol

Fifty rats after 2 weeks of acclimatization were randomly divided into five groups of ten each, as follows: control group: ten rats received saline intraperitoneally. Forty animals received diethylnitrosamine (Den) at a dose of 55 mg/kg body weight twice a week [30. ] then classified into 4 groups as follows: Den-treated group, doxorubicin/Den-treated group: Dox was administered intraperitoneally for 24 h at a dose of 10 mg/kg body weight, twice a week; butanol fraction/Den-treated group: at a dose of 50 mg/kg body weight by oral gavage of butanol fraction intragastrical every day for 4 weeks; ethyl acetate fraction/Den-treated group: at a dose of 50 mg/kg ethyl acetate body weight by oral gavage intragastrical every day for 4 weeks; acacetin 7-*O*-glucoside/Den-treated group: at a dose of 0.44 mg/kg, by oral gavage [31. ]. The total treatment period during the whole experimental study was 6 weeks. All treatments were administered orally.

At the end of the experiment, the rats were euthenized by decaption. Blood and liver samples were collected. The Sera were separated using centrifugation and stored at − 80 °C until analysis. The livers were washed in ice-cold isotonic saline and blotted dry between two filter papers. The portions of liver tissue samples were homogenized in ice-cold water. 1.15% KCl was added to make a (10% w/v) homogenate with a Glass-Col motor-driven homogenizer (USA), and the homogenate was used for the determination of malondialdehyde (MDA). The other portion of the liver tissue was homogenized in ice-cold water. Five percent sulfosalicylic acid to make a (10% w/v) homogenate for the estimation of reduced glutathione (GSH). The sections of the liver were kept in 10% neutral buffer formaldehyde for histological examination.

#### Determination of Liver Toxicity Indices

The serum levels of ALT and AST were determined by a colorimetric method using a Bio-diagnostics kit (AL 1031, AS 1061), while the activity of ALP was performed according to the method of Belfield and Goldberg using the Reactive Gpl kit ref. EZ002LQ-SP.

#### Determination of Pro-inflammatory Markers and Caspase-3 in Liver Tissue

The liver pro-inflammatory markers, AFP and TNF-α, were measured by a colorimetric ELISA kit (Abia Ref. DK.045.01.3 and Sino Gene Clon Ref. SG20127, respectively). Bioassay technology laboratory kits (Ref E0119Ra and Cat. No. BT-AP01203, respectively) were used to estimate Rat IL-1β and Caspase-3.

#### Determination of Oxidative Stress Markers in Liver Tissue

The supernatant of homogenized liver samples was used for the determination of GSH and MDA levels using bio-diagnostics kits (CAT NO. GS 2511 and MD 2529, respectively).

#### Gene Expression Determination of Bax&Bcl2 in Liver Tissue by RT-PCR

Gene expression RT-PCR approximately 30 mg of the liver tissue was stored in an RNA lysis solution at 80 °C until genetic processing. Assessment of Bax and Bcl2 gene expression was done by real-time quantitative reverse transcription PCR (RT-PCR): total RNA was extracted from frozen samples using TRIzol® reagent (Invitrogen, Sigma-Aldrich, St. Louis, MO, USA) according to a standard protocol. The isolated total RNA was converted into complementary DNA (cDNA) using SMARTScribeTM Reverse Transcriptase (Clontech Laboratories, Inc., a Takara Bio Company). RT-PCR was performed using a Real-Time PCR v 7.9 System (DTlite, DNA Technology, LLC, 125Zh Varshavskoe highway, bld. 6, Moscow, Russia, 117587) and SYBR® Green PCR Master Mix (QIAGEN) in a final volume of 25 L with the following thermal cycling conditions: 95 °C for 15 s, followed by 40 cycles of 95 °C for 15 s, 60 °C for 15 s, and 72 °C for 45 s. The sequences of PCR primer pairs used for each gene are shown in Table 1. The ABI Prism sequence detection system software was used to analyze the data, and the PE Biosystems (Foster City, CA) v17 Sequence Detection Software was used to quantify it. The relative expression of the studied genes was calculated using the comparative threshold cycle method. All values were normalized to the GAPDH gene as an invariant endogeneous control (reference gene).

**Table 1:** Primer sequence of genes used in RT-PCR

	Sequence	Accession number
Bax	Forward: 5′GAACCATCATGGGCTGGACA3′ Reverse: 5′TGAGGTTTATTGGCGCCTCC3	XM_032915032.1
Bcl2	Forward: 5′GAACTGGGGGAGGATTGTGG3′ Reverse: 5′ACTTCACTTGTGGCCCAGAT3	XM_034943915.1
GAPDH	Forward: 5′GACAGTCAGCCGCATCTTCT3′ Reverse: 5′GCGCCCAATACGACCAAATC3	XM_003819132.3

#### Histological Examination

Liver tissue embedded in paraffin blocks was sectioned at 5-μm thickness, de-paraffinized, rehydrated, and stained with H&E stain to assess histopathological changes and Picro-Sirius red stain for detection of collagen fibers, and blindly examined for the extent of liver damage under an Axio research microscope (Zeiss) with an attached digital camera (MRC5, Zeiss). Measurement of tissue fibrosis by morphometric analysis using the Image-J software program on Windows 10.

### Statistical Analysis

Statistical analysis was achieved using Graph Pad Prism. Software Inc., Program, version 5.0. The data were presented as mean ± SE, and the level of significance was set at *p* ˂ 0.05. Multiple comparisons were done using one-way ANOVA followed by the Dunnett test as a multiple comparison test.

## Results

### Quantitative Estimation of Total Phenolic and Flavonoid Content

The phenolic and flavonoid content of *T. erecta* L. leaf extract were 78.6 ± 5.15 (gallic acid equivalent) μg/g and 79.0 ± 6.30 (rutin equivalent) μg/g, respectively, as shown in Table 2.

**Table 2:** Quantitative estimation of total phenolic & flavonoid contents in *T. erecta* leaves

*Thunbergia erecta* leaves	Absorbance at 630 nm	Total phenolics (gallic acid equivalent) μg/g
	0.300 (SD ± 0.02)	78.6 μg/g (SD ± 5.15)
	Absorbance at 510 nm	Total flavonoids (rutin equivalent) μg/g
	0.171 (SD ± 0.012)	79.0 μg/g (SD ± 6.3)

### Isolated Compounds Identification

#### Compound 1

Yellowish white amorphous powder (10 mg). Chromatographic properties dark purple fluorescence spot with *R*_*f*_ values; 0.53 and 0.42 in solvent systems in solvent systems BAW (4:1:5 v:v:v) & (15% aqueous acetic acid) dark purple fluorescent under UV light which turned to yellow on exposure to ammonia vapor, or spraying with AlCl3 reagent), and gives positive Molisch’s test. negative ESI-MS/MS Spectrum; showed its molecular ion peak [M-H]^−^ at m/z 593.271 and 201.78, 125.0014 Da. ^1^H NMR (850MHz, DMSO-*d6*); δ_PPM_ 13.6 (1H, s, OH-5), 8.01 (2H, d, *J* = 8.5 Hz, H-2′/6′), 6.91 (2H, d, *J* = 8.5 Hz, H-3′/5′), 6.56(1H, s, H-3), 5.46 (1H, d, *J* = 9.9 Hz, H 1′′), 5.11 (1H, d, *J* = 9.2 HZ, H 1′‵‵), 3.85–3.16 remaining of two sugar protons. ^13^CNMR (213 MHz, DMSO-*d6*) &DEPT.Q 100 MHz); δ_PPM_ 182.7 (C-4), 164.4 (C-2), 159.0 (C-7) 161.6 (C-4`), 159.1 (C-5), 155.6 (C-9), 129.4 (C-2`/6`), 122.0 (C-1`), 116.1 (C-3`/5`), 107.9 (C-6), 105.6 (C-8), 102.9 (C-3), 82.2, 81.2 (C-5″,5‵‵‵), 78.2 (C-3″, 3‵‵‵), 73.8, 72.2 (C-1″, 1‵‵‵), 71.4, 71.0 (C-2″, C-2‵‵‵), 69.5 (C-4″, 4‵‵‵) and 61.7, and 60.3 (C-6″, 6‵‵‵).

#### Compound 2

yellow amorphous powder chromatographic properties dull yellow spots changed to bright yellow with ammonia vapors, green color with FeCl3 and negative Molisch’s test. *R*_*f*_ values; 0.80 and 0.04 in solvent systems BAW (4:1:5 v:v:v) & (15% aqueous acetic acid). ^1^H NMR (400 MHz, DMSO-*d*_*6*_); δ_PPM_ 8.03 (2H, d, *J* = 8.8 Hz, H-2′/H6′), 6.92 (2H, d, *J* = 8.8 Hz, H3′/5′), 6.44 (1H,d *J* = 2 Hz , H8), 6.19 (1H, d, *J* = 2 Hz, H-6).

#### Compound 3

Yellowish white amorphous powder and show purple fluorescence (UV light) and light green color with FeCl_3_ and weak unclear change with ammonia vapors. ^1^HNMR (400 MHz, DMSO-*d6*) δ_PPM_ 8.52 (1H,s, H-2), 7.97 (2H, d, *J* = 9.2 Hz, H-2′/6′), 7.09 (2H, d, *J* = 7.2 Hz, H3′/5′), 6.66 (1H, s, H8), 5.95 (1H,s, H6),4.91 (1H, d, *J* = 7 Hz, H1′′), 3.85 (3 H, s, OCH_3_). **Negative ESI/MS**: m/z 445.19 [M-H]^−^, 281 [M-glucosyl-2H]^−^.

#### Compound 4

Colorless crystalline powder, chromatographic properties, no fluorescence (UV light), give positive Molisch’s test and on TLC gave sharing spot after spraying with 10% (ethanol/H_2_SO_4_) spray reagent, gave reddish violet spot-on TLC with diphenylamine positive ESI-MS/MS: m/z 525.47 [M + 4H + NH_4_]^+^, 365.10, 237.05, and 147.23. ^1^HNMR (850 MHz, DMSO-*d*_*6*_); δ _PPM_ fructose moiety 3.6 (s, H1), 4.19 (d, *J*_3,4_ = 7 Hz, H3), 4.17 (d, *J*_3,4_ = 7 Hz, H4), 3.69 (H5), 3.62 (s, H6) for β-glucose moiety 4.31 (d, *J*_1,2_ = 7.65Hz, H1`β-glucose), 3.25 (br, H3′, β-glucose), 2.96 (t, *J*_3,4_ = 8.5Hz, H4`, β-glucose), 3.70 (m, H5′ β-glucose), 3.72 (brd, H6′ β-glucose). For α-glucose moiety 4.89 (d, *J*_1,2_ = 4.25Hz, H1``α-glucose), 3.15–3.17 (dd, *J*_3,4_ = 9.3, 3.4 Hz, H2`` α-glucose), 3.25 (m, H3`` α-glucose), 2.96 (t, *J* **=** 8.5 Hz, H4′′ α-glucose), 3.70 ( m, H5′′ α-glucose), 3.72 (brd, H6`` α-glucose). ^13^CNMR spectral data (213MHz, DMSO-*d6*): δ_PPM_ for fructose 60.0 (C1), 104.3 (C2), 81.4 (C3), 75.9 (C4), 81.8 (C5), 61.0 (C6), for β-glucose 97.1 (C1`), 72.5 (C2`), 75.4 (C3`), 71.0 (C4`), 75.1 (C5`), 60.6 (C6`) for α-glucose 92.5 (C1``), 71.7 (C2``), 73.7 (C3``), 70.2 (C4``), 74.9(C5``), 68.5 (C6``).

#### Compound 5

**Fig. 1:**
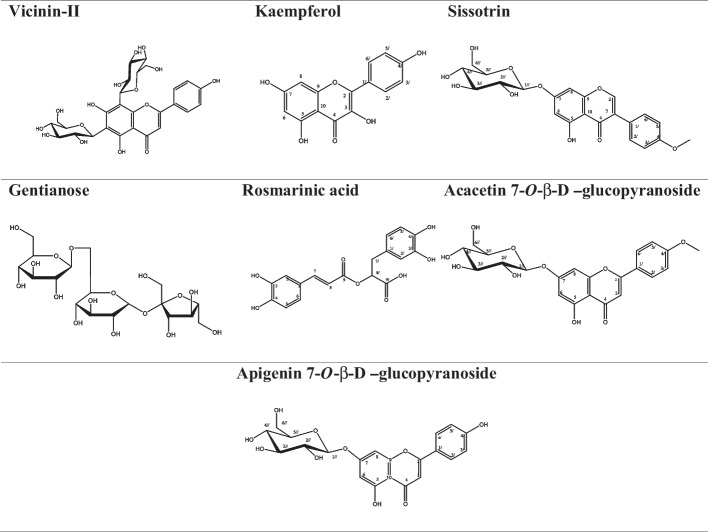
Compounds isolated from *T. erecta* L. leaf extract

Off-white amorphous powder (0.5 g). Chromatographic properties dark purple fluorescence spot with *R*_*f*_ values; 0.63 and 0.79 in solvent systems BAW (4:1:5 v:v:v) & ethyl acetate: methylene chloride:methanol:water (80:20:18:2 v:v:v:v) respectively. Dull yellow florescence (UV light/ammonia vapor), give green color with FeCl3 spray reagent and gives positive Molisch’s test. ^1^H NMR (850MHz, DMSO-*d6*); δ_PPM_ 12.93 (1H, s, OH 5), 8.07 (2H, d, *J* = 8.4 Hz, H-2′/6′), 67.13 (2H, d, *J* = 8.4 Hz, H-3′/5′), 6.97(1H, s, H-3), 6.87(1H, d, *J* = 2.55 Hz, H-8) 6.46 (1H, d, *J* = 2.55 Hz ,H-6), 5.08 (1H, d, *J* = 7.65 Hz, H 1′′), 3.87 (3H, s, OCH3), 3.74–3.17 (may hidden by water signals) remaining of sugar protons. ^13^CNMR (213 MHz, DMSO-*d6*) & DEPT-Q 100 MHz); δ _PPM_ 182.1 (C-4,Q), 163.8 (C-2 ,Q), 163 (C-7,Q), 162.5 (C-5,Q), (C-4′,Q) 161.1, 157 (C-9,Q), 128.5 (C-2′, C-6′, CH), 122.7 (C-1′ Q), 114.68 (C-3′, C-5′, CH), 105.4 (C-10 Q), 103.8 (C-3,CH), 99.8 (C1′′, CH), 99.5 (C-6, CH), 94.9 (C-8, CH), 77.1 (C5′′, CH), 76.4 (C3′′, CH), 73.1 (C2′′, CH), 69.5 (C4′′, CH), 60.5(C6′′ CH2), and 55.6 (OCH3). Figure 1 and Supplementary materials: Fig 1S-14 S,  showed all isolated compounds.

### Biological Results of *T. erecta* Leaf Fractions (Ethyl acetate, Butanol) and Acacetin 7-O-Glucoside

####  Liver Function

Significant increase in liver enzymes (ALT, AST, and ALP) (*p* ≤ 0.05) was observed in the Den group when compared to control group. In contrast, Dox / Den-treated animal caused decrease in their levels when compared to Den-treated rat. Both fraction*s of T. erecta* L. supplementation resulted in a moderate reduction in the activity of ALT, AST, and ALP, while ethyl acetate fraction showed a significant reduction in ALT as compared to the Den-treated group. Acacetin glycoside was able to restore liver enzyme activity as shown in Table 3.

**Table 3:** Effect of *T. erecta* leaves fractions (ethyl acetate, butanol) & acacetin 7-*O*-glucoside on each of the following: liver enzymes

Liver enzymes	Group 1 Control	Group 2 Den [55 mg/kg]	Group 3 Den + DoX [10 mg/kg]	Group 4 Den + EB [50 mg/kg]	Group 5 Den + EE [50 mg/kg]	Group 6 Acacetin 7-*O-*glucoside [0.44 mg/ml]
ALT U/l	19 ± 1.2	29 ± 2.6*	14 ± 1.4^#^	25 ± 1*^#^	16 ± 2.4^#^	18.3 ± 0.33^**#**^
AST U/l	48 ± 0.5	80 ± 0.8*	59 ± 1.8^#^	74 ± 1.5*^#^	74 ± 1.5*^#^	50.5 ± 0.8^**#**^
ALP U/l	102 ± 6	141 ± 2*	120 ± 1.2^#^	137 ± 1.5*^#^	130 ± 2.6*^#^	98 ± 0.57

Data are represented as mean ± SEM (*n* = 6). * *p* ˂ 0.05 vs. normal control, # *p* ˂ 0.05 vs Den, *# > 0.05, *p* ˂ 0.05 vs Den + Dox. Statistical analysis was done using one-way ANOVA followed by the Dunnett test as a multiple comparison test. *ALT*, analine aminotransferase; *AST*, aspartate aminotransferase; *ALP*, alkaline phosphatase; *IL-Iβ*, interleukin 1 beta; Caspase-3, cysteine-aspartic protease-3; *Rat TNF-α*, rat tumor necrosis factor-alpha; *MDA*, malondialdehyde; *GSH*, glutathione; *Dox*, doxorubicin; *EB*, erecta butanol; *EE*, erecta ethylactate. (*significant from -ve control gp &# significant from +ve control)

#### Pro-inflammatory Cytokines and Chemokine’s (IL-1β and TNF-α) and Indicator of Apoptosis Factor (Caspase-3)

Amendment in the level of pro-inflammatory cytokines and inflammatory mediators, specifically, caspase-3, TNF-α, and IL1β, is enlightened in Table 4. Den intoxication markedly enhanced the pro-inflammatory cytokine and inflammatory mediators when compared to normal control. The current study revealed that both fraction*s of T. erecta* L. attenuated the poisonous effect of Den by reducing the activity of enhanced inflammatory mediators. Acacetin glycoside has the most amelioration effect.

**Table 4:** Effect of *T. erecta* leaves fractions (ethyl acetate, butanol) & acacetin 7-*O*-glucoside on each of the following: inflammatory mediators

Inflammatory mediators	Group 1 Control	Group 2 Den [55 mg/kg]	Group 3 Den + DoX [10 mg/kg]	Group 4 Den + EB [50 mg/kg]	Group 5 Den + EE [50 mg/kg]	Group 6 Acacetin 7-*O-*glucoside [0.44 mg/ml]
IL-Iβ pg/ml	146 ± 1	204 ± 0.5^*^	139 ± 1	192 ± 1	186 ± 1	142 ± 3
Caspase-3 pg./ml	0.75 ± 0.3	3.9 ± 0.15^*^	1.15 ± 0.05	2.0 ± 0.1	2.4 ± 0.1	1.8 ± 0.1
Rat TNF-α ng/ml	38.5 ± 0.5	48.3 ± 1.3^*^	42.7 ± 0.9	56.6 ± 0.6	59 ± 0.6	43 ± 1

Data are represented as mean ± SEM (*n* = 6). *p < 0.05 vs. normal control, #*p* <0.05 vs Den, *#*p* < 0.05 vs Den + Dox. Statistical analysis was done using one-way ANOVA followed by the Dunnett test as a multiple comparison test. *ALT*, analine aminotransferase; *AST*, aspartate aminotransferase; *ALP*, alkaline phosphatase; *IL-Iβ*, interleukin 1 beta; Caspase-3, cysteine-aspartic protease-3; *Rat TNF-α*, rat tumor necrosis factor-alpha; *MDA*, malondialdehyde; *GSH*, glutathione; *Dox*, doxorubicin; *EB*, erecta butanol; *EE*, erecta ethylactate. (* significant from -ve control &# significant from +ve control)

#### Oxidative Stress Markers (MDA, Reduced Glutathione GSH)

Our data shows that DEN administration was found to diminish the hepatic antioxidants GSH but markedly increase in the MDA level when compared to the control group. However, both fraction*s of T. erecta* L. caused a significant amelioration in the GSH content and MDA level. Acacetin glycoside supplementation has the same effect of ethyl acetate fraction (Table 5).

**Table 5:** Effect of *T. erecta* leaves fractions (ethyl acetate, butanol) & acacetin 7-*O*-glucoside on each of the following: oxidative stress markers

Oxidative stress markers	Group 1 Control	Group 2 Den [55 mg/kg]	Group 3 Den + DoX [10 mg/kg]	Group 4 Den + EB [50 mg/kg]	Group 5 Den + EE [50 mg/kg]	Group 6 Acacetin 7-*O-*glucoside [0.44 mg/ml]
MDA nmol/g tissue	24 ± 0.5	50 ± 0.9^*^	30 ± 0.4^#^	34 ± 0.7^#^	33.6 ± 0.5^#^	36 ± 1.5
GSH nmol/g tissue	13 ± 0.2	6.5 ± 0.2^*^	8.4 ± 0.05^#^	7.8 ± 0.05^#^		10.6 ± 0.3

Data are represented as mean ± SEM (*n* = 6). *p <0.05 vs. normal control, #*p* <0.05 vs Den, *#*p* <0.05 vs Den + Dox. Statistical analysis was done using one-way ANOVA followed by the Dunnett test as a multiple comparison test. *ALT*, analine aminotransferase; *AST*, aspartate aminotransferase; *ALP*, alkaline phosphatase; *IL-Iβ*, interleukin 1 beta; Caspase-3, cysteine-aspartic protease-3; *Rat TNF-α*, rat tumor necrosis factor-alpha; *MDA*, malondialdehyde; *GSH*, glutathione; *Dox*, doxorubicin; *EB*, erecta butanol; *EE*, erecta ethylactate. (* significant from -ve control &# significant from +ve control)

#### Alpha-Fetoprotein (AFP)

Activity of the serum alpha-fetoprotein (AFP) level was significantly increased in Den-treated group when compared to the normal group (Table 6). Significant decline in the serum AFP level was observed in the treatment groups when compared with the DEN group.

**Table 6:** Effect of *T. erecta* leaves fractions (ethyl acetate, butanol) & acacetin 7-*O*-glucoside on each of the following: Bax and Bcl2 gene expression

RT-PCR (gene expression)	Group 1 Control	Group 2 Den [55 mg/kg]	Group 3 Den + DoX [10 mg/kg]	Group 4 Den + EB [50 mg/kg]	Group 5 Den + EE [50 mg/kg]	Group 6 Acacetin 7-*O-*glucoside [0.44 mg/ml]
Bax	0.003 ± 1	3.6 ± 0.08^*^	2.9 ± 0.08^*#^	1.86 ± 0.05*#	1.83 ± 0.06*	1.4 ± 0.03
Bcl2	1 ± 0.005	0.2 ± 0.05^*^	0.7 ± 0.03^*#^	1.8 ± 0.05^#^	1.56 ± 0.03^#^	2.3 ± 0.17
Bax/Bcl2	1 ± 0.006	14 ± 2.1^*^	3.8 ± 0.25^#^	0.99 ± 0.003^*#^	1.106 ± 0.02^*#^	0.6 ± 0.025

Data are represented as mean ± SEM (*n* = 6). *p <0.05 vs. normal control, #*p* <0.05 vs Den, *#*p* <0.05 vs Den + Dox. Statistical analysis was done using one-way ANOVA followed by the Dunnett test as multiple comparison test. *ALT*, analine aminotransferase; *AST*, aspartate aminotransferase; *ALP*, alkaline phosphatase; *IL-Iβ*, interleukin 1 beta; Caspase-3, cysteine-aspartic protease-3; *Rat TNF-α*, rat tumor necrosis factor-alpha; *MDA*, malondialdehyde; *GSH*, glutathione; *Dox*, doxorubicin; *EB*, erecta butanol; *EE*, erecta ethylactate. (* significant from -ve control &# significant from +ve control)

#### On Bax and Bcl2 RT-PCR Gene Expression

The diethylnitrosamine-treated group revealed significant increase in expression of Bax (apoptotic gene) but significant decrease in Bcl2 (anti-apoptotic gene) gene when compared to the control group, while there were significant differences between all treated groups, but the treatment with *T. erecta* L. Ethyl acetate fraction was more effective in decreasing the expression of Bax gene than other treatments. And, the most effective fraction was acacetin 7-*O*-β-glucopyranoside in increasing the expression of Bcl2 gene (Table 7).

**Table 7:** Effect of *T. erecta* leaves fractions (ethyl acetate, butanol) & acacetin 7-*O*-glucoside on each of the following: tumor necrosis factor AFP IU/I

	Group 1 Control	Group 2 Den [55 mg/kg]	Group 3 Den + DoX [10 mg/kg]	Group 4 Den + EB [50 mg/kg]	Group 5 Den + EE [50 mg/kg]	Group 6 Acacetin 7-*O-*glucoside [0.44 mg/ml]
AFP IU/I	1.8 ± 0.0#	3.8 ± 0.1#	3.6 ± 0.2#	3.1 ± 0.1#*	4.3 ± 0.2*	0.5 ± 0.1

Data are represented as mean ± SEM (n = 6). *p <0.05 vs. normal control, #*p* <0.05 vs Den, *#*p* <0.05 vs Den +Dox. Statistical analysis was done using one-way ANOVA followed by the Dunnett test as multiple comparison test.(* significant from -ve control &# significant from +ve control)

#### Histopathological Examination

**Fig. 2:**
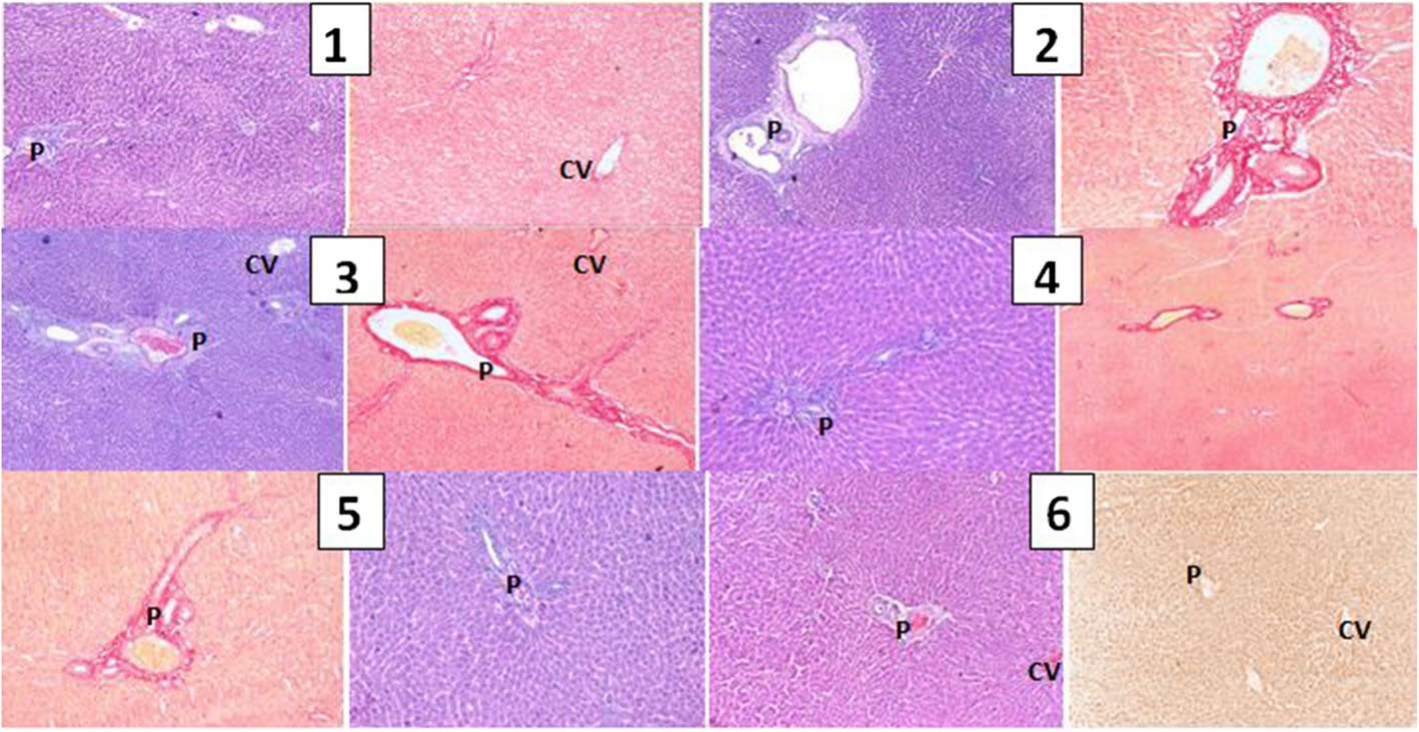
(1) Sections in control no infected mice with normal hepatic lobular architecture ((H&E stain and Picro-Sirius red stains, × 100). (2) Section in control non-treated liver tissue showing normal hepatic architecture with no excess deposition of fibrous tissue (H&E stain and Picro-Sirius red stains, × 100). (3) Section in liver of group (2) showing deposition of fibrous tissue, mostly in the portal tract (H&E stain and Picro-Sirius red stains, × 200). (4) Sections in liver of group (3 and 4) showing deposition of variable amounts of fibrous tissue in hepatic parenchyma and the portal tract (H&E stain and Picro-Sirius red stains, × 200). (5) Liver section in group (5) showing nearly preserved hepatic lobular architecture, with no evidence of fibrosis or nodular transformation (H&E stain and Picro-Sirius red × 100). (6) Liver section in group 6 showing nearly preserved hepatic lobular architecture, with no evidence of fibrosis or nodular transformation (H&E stain, Picro-Sirius red X100). No other significant histopathological changes were detected within different groups concerning hepatocytic degeneration or lobular inflammation

Histopathological examination of liver sections from different groups showed the highest score of liver fibrosis in the control-positive group with portal tract deposition of collagen fibers (group 2) [Table 8 and Fig. 2 (2–6)], with high significant difference in comparison with the negative control group (group 1) [Fig. 2 (2)]. All treated groups (3–6) [Fig. 2 (4–6)] showed significantly higher fibrosis score in the form of portal tract deposition of collagen, compared to the negative control group (1) and significantly lower fibrosis scores compared to the positive control group 2. Group 6 showed the least mean percentage of fibrotic area.

**Table 8:** Effect of *T. erecta* leaves fractions (ethyl acetate, butanol) & acacetin 7-*O*-glucoside on each of the following: histopathological examination

	Group 1 Control	Group 2 Den [55 mg/kg]	Group 3 Den + DoX [10 mg/kg]	Group 4 Den + EB [50 mg/kg]	Group 5 Den + EE [50 mg/kg]	Group 6 Acacetin 7-*O-*glucoside [0.44 mg/ml]
Score of liver fibrosis	1.13 ± 0.2	9.86 ± 1.2*	1.9460 ± 0.35 #	4.34 ± 0.81*#^	4.3 ± 0.16*#^	1.08 ± 0.43^#^

Histological assessment of percentage of tissue fibrosis in liver sections in the four examined treated groups (*Den.*, diethylnitrosamine; *dox*, doxorubicin; *EE*, *Thunbergia erecta* ethyl acetate extract; *EB*, *Thunbergia erecta* butanol extract; and acacetin 7-*O*-glucopyranosid) (represent the mean ± SEM) showed a significantly higher value (*p* < 0.001) vs. the control (*p* < 0.001) vs Den. (* significant from -ve control &# significant from +ve control), (^) indicate significance from doxorubicin

.

## Discussion

### Compound 1

Based on the chromatographic properties in response toward different spray reagent as well as comparison with authentic samples and resistance to complete acid hydrolysis [32. ], di-C-glycoside apigenin was expected to be. ^1^HNMR spectral data showed A2B2 type of two ortho-coupled protons in aromatic region at δ_PMM_ 8.01 and 6.91 (each 2H, d, *J* = 8.5 Hz) assignable to H-2′/6′ and H-3′/5′, respectively in ring B in addition to one singlet proton at 6.56 ppm characteristic for H-3 and the δ_H_ and *J* value of H1‵‵ and H1‵‵‵ doublet at 5.46 and 5.11 (*J* = 9.9 and 9.2Hz) respectively guided us to characterize sugar part as bi-C-hexose. ^**13**^**C-NMR** exhibited 13 carbon resonances characteristic for apigenin aglycone particularly C-4, (C-2`/6`), and (C-3`/5`) at δ_C_182.7, 129.4, and 116.1 respectively can be considered key signals of aglycone moiety; in aliphatic region, 12 intrinsic carbon resonances were assigned for a bi-C-β-glucopyranoside particularly those at δ_C_ 82.2, 81.2 assigned for 5″, 5‵‵‵, 78.2 assigned for 3″, 3ʹʹʹ, 73.8, 72.2 for 1″, 1ʹʹʹ and the downfield shift (≈ ∆ + 10 ppm) of C-8 and C-6 at δ _C_ 105.6 and 107.9 respectively relative to its normal position of apigenin aglycone was a diagnostic evidence for the C-glycosidation at C-8 and 6. **Negative ESI-MS** showed its molecular ion peak at m/z 593 corresponding to its molecular weight 594 of di-hexoside structure corresponding to C_27_H_30_O_15_ which confirm C-type glycoside linkage [33. ], to finally identified as 6,8-di-*C*-*β*-d-glucopyranoside apigenin vecinin-II (Fig. 1 & 1-3S).

### Compound 2

A_2_B_2_ spin coupling system of two ortho-coupled protons in aromatic region at *δ* = 8.06 and 6.9 (each 2H, d, *J* = 8.8 Hz) assignable to H-2′/6′ and H-3′/5′, respectively, of 1.4 di-substituted β-ring were as the second one explained as AM of two meta-coupled protons at *δ* = 6.44 and 6.19 (each 1H, d, *J* = 2 Hz) assigned to H-8 and H-6, respectively. The spectrum further revealed absence of H-3 proton indicates 3,5,7,4‵-tetrahydroxyflavone (kaempferol) (Fig. 1 & 4S) [34. ].

### Compound 3

showed in aromatic region an AX spin coupling system of two ortho doublets, 2H/each, characteristic of 1,4 di-substituted B-ring. The relative downfield location of the X-type doublet (H3′/5′) at δ_H_ 7.09 (~ ∆ + 0.2 ppm) was indicative for 4′-*O*-methoxy, which was assigned as singlet at 3.85. The isoflavone was also supported by the characteristic location of H-2 resonance at about δ_H_ 8.52 ppm. As well as the downfield shift of H-8 proton (≈ ∆ + 0.25 ppm) together with the presence of a β-anomeric proton signal at 4.91 (H1′′), were two evidence of the attachment of β-glucopyranoside moiety on OH-7 as *O*-glycoside. Negative ESI/MS showed a molecular ion peak at m/z 445.19 [M-H]^−^ corresponding to mol. weight 446 assigned for molecular formula C_22_H_22_O_10_, together with other fragment ion at 281[M-hexose-2H]^−^ assigned for a glycone [35. ] to confirm the structure as sissotrin (Fig. 1 & 5-6S).

### Compound 4

from chromatographic properties expected to be sugar with no florescence under UV light then gave black spot-on TLC after spraying with (10–15 % ethanol/H_2_SO_4_) at 120 °C. In addition to positive Molisch’s test. ^1^H and ^13^CNMR allowed us to identify a trisaccharide containing two units of glucose (G1and G2) and one fructose unit (F). The two doublets at δ_H_ ppm 4.89 (d, *J* = 4.2Hz) and 4.31 (d, *J* = 7.65Hz), the chemical shift of two signals, their multiplicity (doublet), and coupling constant value (*J*) allowed to attribute them to the anomeric protons of two hexose moieties, the first one in α (G1) and the second in β configuration (G2). ^13^CNMR spectrum confirmed this hypothesis by showing three signals in the anomeric region at δ_C_ ppm 104.34 which representative to C-2 of fructose, 97.15 (G2-C1), and 92.50 (G1-C1). All NMR data lead to conclusion that G1 is linked through α glycoside bond while G2 through β one, suggestion a trisaccharide [36. ] and depending on its positive ESI/MS the molecular weight deduced as 504 mu characteristic for C_18_H_32_O_16_ which representative for oligosaccharide formed of three hexose moieties from previous NMR expected as α-G1, β-G2, and β-F, and from the fragment ion peak at m/z 365mu [M + NH4 + 4H-hexose]+ characteristic for C_12_H_22_O_11_ which representative for sucrose moiety, confirmed by 1HNMR spectrum, α-G1 at 4.89 (1H, d, *J* = 4.2Hz), in addition to characteristic chemical shift of F-H-3′, F-H-4′ resonances at δ_H_ 4.19 (d, *J* = 7Hz) key proton signals for sucrose, in addition to finger print region for sucrose in ^13^CNMR at δ_C_ 92.50,104.34, 60.01, and 61.07assigned for C1-α-G1, C2-F, C1-F, and C-6-F respectively [37. ]. The downfield shift about (≈ + 7ppm) of C6-G1 sucrose at 68.51 ppm confirm the attached C1-β-G2-on C6-αG1 [38. ] from previous data and reported data [38. ] the present compound identified as gentianose (Fig. 1 & 7-9S).

### Compound 5

from its chromatographic properties may be flavone in nature. ^**1**^**H NMR** showed signals for an exchangeable proton at δ_H_ 12.93 (1H, s-5-OH ), A2B2-spin coupling system protons at δ 8.07 and 7.13 (d, *J* = 8.4 Hz) assigned for (H-2′/H-6′) and (H-3′/H-5′) of B-ring respectively, two meta doublet protons at δ_H_ 6.87 and 6.46 with *J* value 2.55 Hz assigned for H-8, H-6 respectively on A-ring, together with an olefinic protons at δ_H_ 6.97 singlet as signal for H-3 on a flavone C-ring in addition, signal at δ_H_ 5.08 (1H, d, *J* = 7.65 Hz) assigned for β glucopyranosyl anomeric proton (H-1′′). Finally presence of s at δ_H_ 3.87 characteristic for the presence of OCH_3_. And from ^**13**^**C NMR**, the spectrum showed 13 carbon resonance agreement with apigenin aglycone; the most characteristic signals were 182.1, 163.8, 163.0, 162.5, 161.1, 157.0, 122.7, and 105.4 assigned for quaternary C-4, C-2, C-7, C-4′, C-5, C-9, C-1′, and C-10 respectively and olefinic carbon resonance at δ_C_ 128.5, 114.6, and 103.8 assigned for C-2′/6′, 3′/5′, and C-3 respectively to confirm the aglycone as apigenin and the presence of carbon resonance at δ_C_ 55.6 characteristic for OCH3 at C4′-methyl ether apigenin which is known as acacetin and the value of anomeric carbon at δ_C_ 99.8 and the carbon resonance of remaining sugar agreement with β-d-glucopyranoside and sugar part attached to C-7 confirmed by the downfield shift of H-8 and H-6 approximately (≈ ∆ + 0.2–0.4 ppm) and by the value of C-7 in ^13^C NMR spectrum, and the data was agreed with data published by [39. , 40. ]. Compound 1 was identified as 4′ methyl ether apigenin 7-*O*-β-d-glucopyranoside which known as acacetin 7-*O*-β-d-glucopyranoside (Fig. 1 and 10-11S).

**Apigenin 7-*O*-glucopyranoside** and **rosmarinic acid** were previously isolated from *T. erecta* aerial parts and identified according to Refaey et al. (2021) [41. ].

### Biological Study

The study aims to evaluate the antioxidant, anti-inflammatory, anti-apoptotic, and antifibrogenic effect of *T. erecta* L. ethyl acetate, butanol fractions, and the major isolated metabolite; acacetin 7-*O*-glucopyranoside against diethylnitrosamine induced liver toxicity. The polyphenolic compounds and flavonoids are identified in *T. erecta* L. and the presence of these biomolecules in erecta leaves makes them a potential bioantioxidant source. A chronic hepatocellular injury is the main cause of liver fibrosis, a serious condition with considerable morbidity and mortality. Patient mortality is increasing with liver fibrosis because it manifests a variety of pathological conditions, sometimes leading to liver cancer [42. ]. Hepatocellular damage is caused by oxidative stress, chronic inflammation, and cellular proliferation caused by nitrosamines, which are associated with liver fibrosis [43. ]. Furthermore, Den changes the expression of enzyme markers in the serum [44. ] and in tissues affected by hepatocyte damage [45. ]. In this study, the researchers noted that hepatic cellular injuries attributable to a number of pathological conditions such as hepatitis caused the elevation of these enzymes. According to previous studies, Den-induced liver injury is associated with increased activity of these enzymes [46. ]. Reactive oxygen species (ROS), generated from oxidative stress, are responsible for contributing to fibrosis, a significant factor in chronic disease [42. ]. Hepatocellular fibrosis induced by Den results in significant increases in inflammatory markers such as interleukin-1 beta (IL-1β) and tumor necrosis factor-alpha (TNF-α) [45. ]. Accordingly, macrophages and neutrophils constituting the liver fibrosis pathogenic cells are thought to release pro-inflammatory cytokines (IL-1β and TNF-α) upon activation [41. ]. Caspases 3 are implicated in the mediation of nuclear apoptosis, including chromatin condensation and DNA fragmentation, as well as bleeding in cells [47. ]. The present study showed that Den has been exhibiting an increase in the expression of the proapoptotic gene Bax, while Bcl-2 expression has declined [27. ]; as a result, the ratio of Bax/Bcl-2 surged dramatically. These results demonstrate that Den induces intrinsic apoptosis during hepatocellular fibrosis as well as facilitates the removal of transformed cells through targeting the Bcl-2 family members [24. , 25. ]. In our study, it was demonstrated that *T. erecta* L. bioactive constituents initiate cell proliferation and survival possible by overexpressing Bcl2—this is attributed to phenolic acids and flavonoids, which have anti-apoptotic properties as has been described previously [48. ]. Due to the presence of hydroxyl groups inside their aromatic rings, phenolic phytoconstituents have strong antioxidant properties [49. ]. Acacetin 7-*O*-glucopyranoside is the most active ingredient isolated from this plant and it provides powerful healing properties. Our current study showed that Den treatment resulted in reduced GSH levels and higher MDA levels in liver tissue of rats diethylnitrosamine causes fibrotic lesions to form in the liver during oxidative damage [44. ]. The antioxidant effect of *T. erecta* L. arises from its antioxidants content as quercetin-3-*O*-xyloside [48. ], kaempferol [50. ], and acacetin7-*O*-glucoside [51. ] which protect the cells from oxidative damage. Many signaling pathways are blocked by flavonoids, resulting in reduced proliferation, angiogenesis, and metastasis [52. ]. A marked reduction in inflammatory cytokine biomarkers (interleukin 1β & TNF-α) was seen in the treated groups (EE, EB, & acacetin 7-*O*-glucoside). As a result of this inhibition, hepatic fibrosis and hepatocellular toxicity associated with Den may have been reduced. In conjunction with these findings, recent studies have shown that herbal bioactive molecules can be effective in preventing liver fibrosis [53. , 54. ]. Histopathological evaluation of H&E and Picro-Sirius red staining showed that from normal control: polyhedral hepatocytes and nuclei were round, prominent nucleoli, arranged around the central vein in a radial pattern with no vacuolated cytoplasm, dispersed chromatin and separated by normal sinusoids, histopathological examination of H&E for routine morphological evaluation, and Picro-Sirius red staining for fibrosis scoring and estimation showed that normal control: conventional hepatic lobular architecture in the form of polyhedral hepatocytes with nuclei were round, arranged around the central vein in a radial pattern with no vacuolated cytoplasm, separated by normal sinusoids. Den treatment induced histopathological damage and increased collagen deposition. With disordered lobular structure, congestion of the centrilobular vein, appearance of foci of steatosis, proliferation of Kupffer cells, dilatation of sinusoids, and infiltration of inflammatory cells. In addition, fragmented chromatin was prevalent in the hepatocytes of intoxicated group by diethylenediamine. While doxorubicin inhibits liver fibrosis induced by Den, and treatment with EE, EB, and acacetin7-*O*-glucoside markedly alleviated and achieved marked improvements in reversing this pathological injury the Den induced in the rats’ livers ( reduction in fibrosis scoring). Furthermore, chromatin fragmentation was much less or absent in the Den-treated DoX. EE, EB, and acacetin 7-*O*-glucoside rats. This histopathological analysis is in accordance with the results of the biochemical diagnostic indicators. This could be due to the observed ability of extracts to recover endogenous antioxidant mechanisms, resulting in the free radicals being scavenged to allow hepatocyte regeneration. This antioxidant effect breaks the vicious cycle of continuous inflammation fibrosis resulting from the continuous activation of KCs, causing a release of pro-inflammatory factors and aiding fibrosis.

## Conclusion

 The present study revealed that acacetin 7-*O*-β-glucopyranoside isolated from *T. erecta***L**. leaf extract, ethyl acetate, and butanol fractions have beneficially hepatoprotective and antifibrotic impact against oxidative injury induced by diethylnitrosamine.* T erecta* L. has a defensive outcome on hepatic fibrosis may be due to its free radical scavenging, antioxidant, and anti-inflammatory effects that are attributed to polyphenolic contents, especially acacetin glucoside. These outcomes may be valuable in developing novel hepatic fibrosis prevention approaches.

### Supplementary Information

Below is the link to the electronic supplementary material.

Supplementary file1 (DOCX 723 KB)

## Data Availability

The data and materials are included in this published article.
