# Supplementary material for: Ameliorative Effects of Thunbergia erecta L. Leaves Against the Initiation of Hepatocarcinogenesis Induced by Diethylnitrosamine in the Rat Model
Source: Appl Biochem Biotechnol. 2023 Jan 28;195(10):5881–902. doi: 10.1007/s12010-022-04292-x (PMC10511368; doi:10.1007/s12010-022-04292-x)
Supplement: Supplementary file 1 — Supplementary file1 (DOCX 723 KB) [file 12010_2022_4292_MOESM1_ESM.docx]

Supplementary data

**Ameliorative Effects of *Thunbergia erecta* L. Leaves Against the Initiation of Hepatocarcinogenesis Induced by diethyl nitrosamine in Rat Model**

**Running Title: Effect of *Thunbergia erecta* L. leaf extract in liver toxicity.**

**Fatma Sayed Abdel-Aal Farag^1*^, Hend Mohamed Anwar^2^, Tarek Aboushousha^3^, Hala Sh. Mohammed^1^, and Lotfi Diab Mousa Ismail^4^**

^1*^ PhD Pharmacognosy and Medicinal Plants Department, Faculty of Pharmacy (Girls), Al-Azhar University, Cairo 11651, Egypt, Email: [fs92020@gmail.com](mailto:fs92020@gmail.com).

^1^Associate Professor, Pharmacognosy and Medicinal Plants Department, Faculty of Pharmacy (Girls), Al-Azhar University, Cairo 11651, Egypt, Email: [halash1977@hotmail.com](mailto:halash1977@hotmail.com)

**^2^** PhD, Department of Biochemistry, National Organization for Drug Control & Research, Cairo, Egypt**.** E.mail**:** [hend.hassan@outlook.de](mailto:hend.hassan@outlook.de) ,

**^3^** Professor, Department of Pathology, Theodor Bilharz Research Institute, Kornaish El-Nile, Warrak El-Hadar, Imbaba (P.O. 30), Giza 12411, Egypt

Email: [t.aboushousha@tbri.gov.com](mailto:t.aboushousha@tbri.gov.com)

^4^ Professor, Pharmacognosy and Medicinal Plants Department, Faculty of Pharmacy (Boys), Al-Azhar University, Cairo 11651, Egypt, Email: [lotfydiab@yahoo.com](mailto:lotfydiab@yahoo.com)

**Corresponding author:**

**Dr. Fatma Sayed Abdel-Aal Farag**, Pharmacognosy and Medicinal Plants Department, Faculty of Pharmacy (Girls), Al-Azhar University, Cairo 11651, Egypt, Email: fs92020@gmail.com

Mobile: 01095280710

ORCID ID: 0000-0002-0842-0259

**Abstract**

Background: *Thunbergia erecta* L. contains cytotoxic and liver-protective compounds.

Objective: *T. erecta* L. leaves were analyzed for total phenolic and flavonoid content and attenuation of diethyl nitrosamine (Den)-induced liver cancer in an experimental model of Wistar rats.

Methods: *T. erecta* L. leaves were macerated in 70% aqueous ethanol before being fractionated using ethyl acetate (9.3g), and butanol (12.7g). Column chromatography and solid phase extraction (SPE) were used to chromatograph ethyl acetate and butanol fractions; seven compounds were isolated, and their structures were identified based on spectral data and comparisons to published data. Sixty rats were divided into six groups (ten each) to evaluate the attenuation of diethyl nitrosamine (Den) by ethyl acetate and butanol fractions of *T. erecta* L. leave*:* control group, Den group, Doxorubicin/Den-treated group, *T. erecta* L. butanol fraction/Den-treated group, and isolated Acacetin7-*O*-glucopyranoside/Den-treated group. The liver enzymes and pro-inflammatory biomarkers were used to estimate the liver enzymes. Also, the liver tissues were collected to determine the oxidative stress markers , gene expression, and finally histopathological examination.

Results: In *T. eracta* L. leaves, total phenolic and flavonoid contents scored 78.6 ± 5.15 (gallic acid equivalent) µg/g and 79.0± 6.3 (rutin equivalent) µg/g respectively. vicenin-II (1), kaempferol (2), biochanin A 7-*O*-β-D-glucopyranoside (sissotrin) (3), and gentianose (4) acacetin 7-*O*-β-D-glucopyranoside (5), apigenin 7-*O*-β-D-glucopyranoside (6), rosmarinic acid (7), were isolated and identified. a significant increase in the levels of liver enzymes, AFP and TNF-ἁ. This was conveyed by a significant increase of IL-1 and caspase-3, elevation of MDA and reduction of GSH, and suppression of Bcl2 and elevation of Bax expression. All parameters in the butanol, ethyl acetate fractions, and isolated acacetin 7-*O*-β-glucopyranoside (major constituents) of *T. erecta* L. were significantly improved to values close to those of the control group.

Conclusion: *T. erecta L.* supplementation successfully decreased the harmful effects of Den. It was increased hepatic antioxidant capacity by increasing reduced glutathione (GSH) efficiency and decreasing malondialdehyde (MDA) levels. Because of its chemo preventive impact on liver cancer, it might be employed as a novel alternative technique.

Key words, Thunbergia, apoptosis, caspase, acacetin, phenolic and diethyl nitrosamine.

**Appendix**

| Item | Subject |
| --- | --- |
| Fig.1(S) | ESI-MS/MS spectrum of Vecinin-II |
| Fig.2(S) | ^1^HNMR (850MHz, dmso*-d6*) spectrum of Vecinin-II |
| Fig.3(S) | ^13^CNMR (213MHz, dmso*-d6)* spectrum of Vecinin-II |
| Fig.4(S) | ^1^HNMR(400MHz, dmso*-d6*) spectrum of kaempferol |
| Fig.5(S) | Negative ESI/ MS of Sissotrin |
| Fig.6(S) | ^1^HNMR (100MHz, dmso*-d6*) spectrum of Sissotrin |
| Fig.7(S) | Positive ESI-MS/MS spectrum of Gentianose |
| Fig.8(S) | ^1^HNMR (850MHz, dmso-*d6*) spectrum of Gentianose |
| Fig. 9(S) | ^13^CNMR(213MHz, dmso-*d6*) spectrum of Gentianose |
| Fig. 10(S) | ^1^HNMR (850MHz, dmso*-d6*) spectrum of acacetin 7-*O*-β-D-glucopyranoside |
| Fig. 11(S) | ^13^CNMR (213MHz, dmso-*d6*)Spectrum of acacetin 7-*O*-β-D-glucopyranoside |
| Fig. 12(S) | Apigenin 7-*O*-β-D –glucopyranoside |
| Fig. 13 (S) | Apigenin 7-*O*-β-D –glucopyranoside |
| Fig. 14 (S) | Rosmarinic acid |
| S1 | Results of isolated compounds 6 and 7 |
| S2 | Structure elucidation of isolated compounds 6 and 7 |


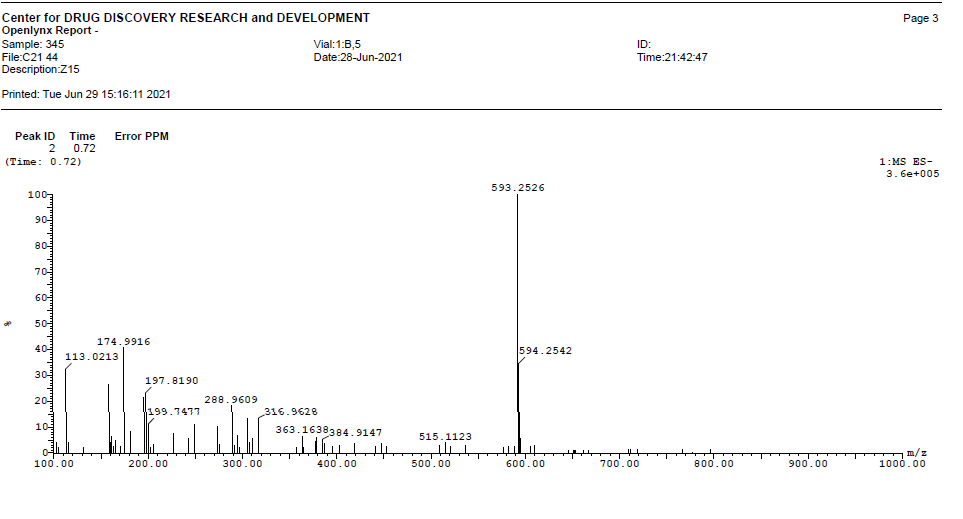


[M-H]^-^

Fig.1(S)

Fig.2(S)

5.1 d,

*J* =9.2Hz (H-1′′′)


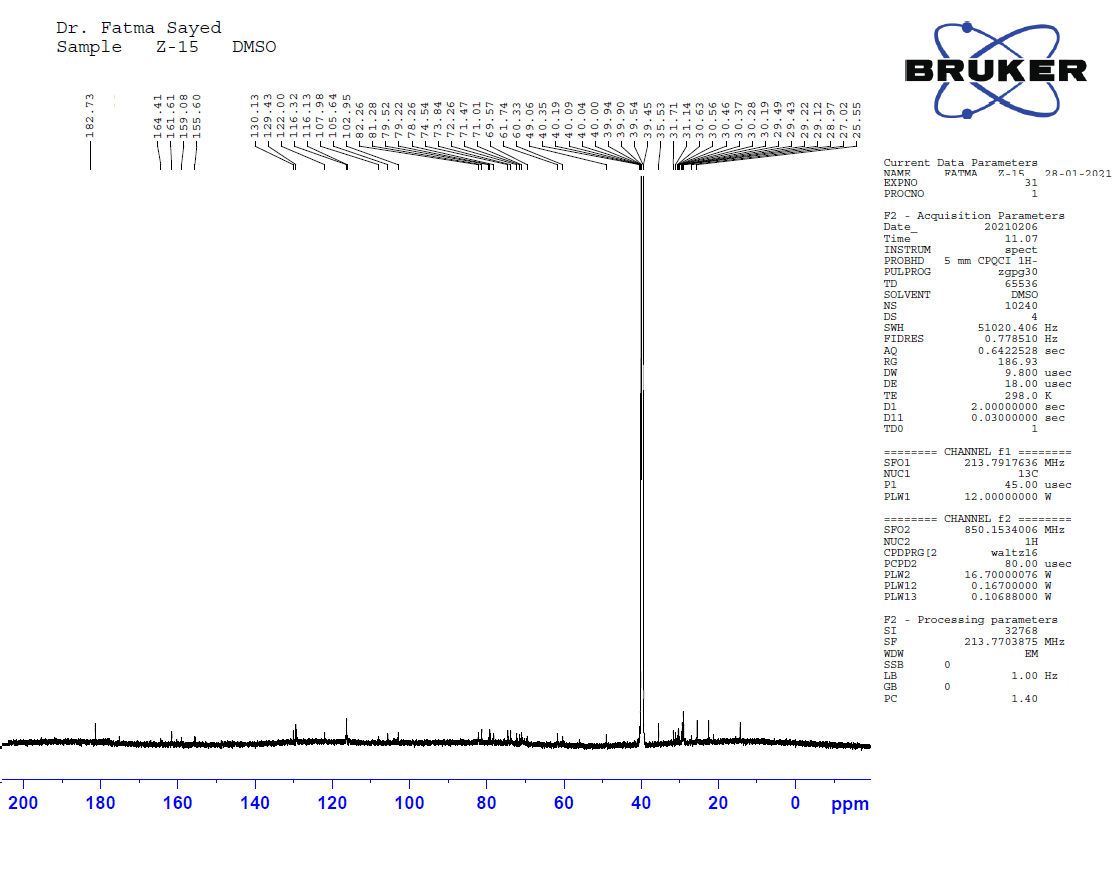
 Fig.3(S)

Fig.4(S)


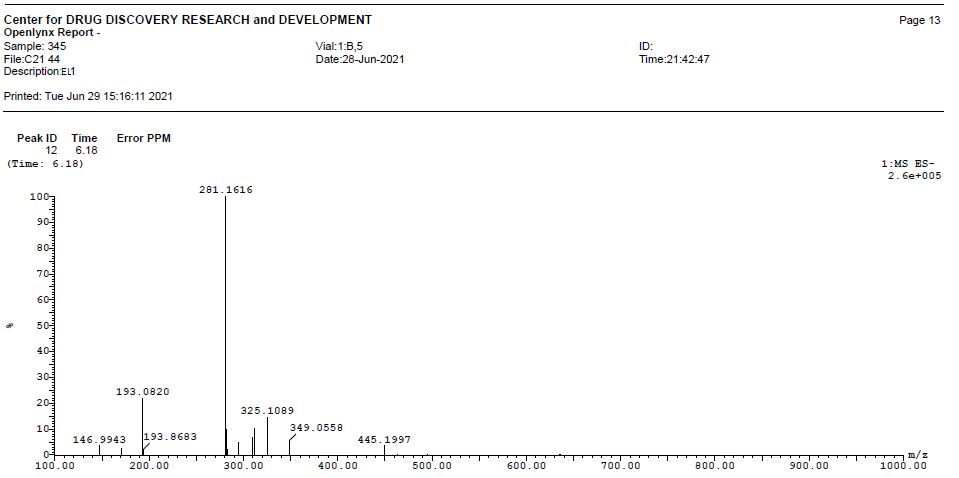


Fig.5(S)

Fig.6(S)


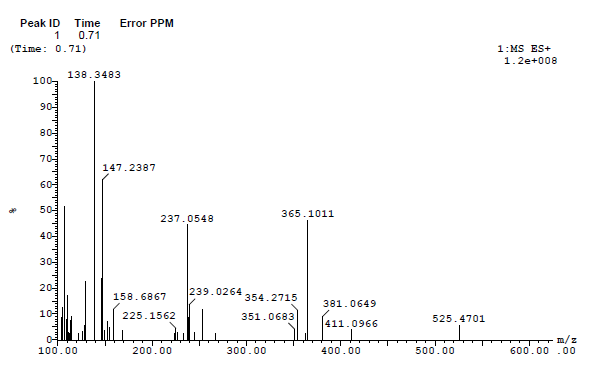


Fig.7(S)

 Fig.8(S)

Fig.9(S)

 Fig. 10(S)

Fig. 11(S)

**S1: Results of compound 6 and 7**

**Compound 6:** ^1^H NMR(400 MHz, DMSO-*d_6_*) :δ 12.97 (1H , s, OH -5 ) , 7.96 (2H ,d , *J* = 8.8 Hz , H-2′/6′) , 6.94 (2,d , J = 8.8 HZ , H-3′/5′) ,6.87 (1H,s, H-3), 6.84(1H, d , *J* = 2.08 Hz, H -8) 6.45 (1H, d , *J* = 2.08Hz, H-6 ) , 5.07 ( 1H, d , *J* = 7 Hz ,H 1′′ ), 3.74-3.17 (hidden by water signals ) remaining of sugar protons. ^13^ C NMR(100 MHz, DMSO-*d_6_*) :δ PPM 182.4 (C-4), 164.7 (C-2) , 162.9 (C-7) , 161.5 (C-5& C-4′) , 157.4 (C-9) ,129 (C -2′ ,C-6′) ,121.4( C-1′) , 116.4(C -3′ ,C-5′) , 104.2 (C-10) ,103.5 (C -3) ,100.0(C1′′) ,99.9 (C-6), 95.3 (C-8) , 77.6 (C5′′), 76.8 (C3′′) , 73.5 (C2′′), 70.0 (C4′′) , 61.06 (C6′′).

**Compound 7:** ^1^H NMR(850 MHz, DMSO-d6) :δ 7.01 (1H ,d , *J* = 1.7 Hz, H-2) , 6.73 (1H,d , *J* = 8.5 Hz , H-5), 6.93(dd, *J* =7.65,1.6Hz, H-6), 7.32 (2H ,d , *J* = 16.15 Hz, H-7) , 6.15(1H, d , *J* = 16.15Hz, H -8), 6.64 (1H ,d , *J* = 1.7 Hz, H-2׳), 6.58 (1H, d , *J* = 8.5Hz ,H -5׳ ), 6.47(1H, d , *J* = 7.65,1.6Hz, H -6׳ ), 2.99 (1H, dd , *J* = 14.45,1.7Hz, H -7׳ ), 5.6 (1H, dd , *J* = 12.75Hz, unresolved H -8׳ )

**S2: Discussion of compound 6 and 7**

From its chromatographic properties, **compound (6)** is flavone glycoside and by comparison with^1^HNMR and ^13^CNMR spectra of compound (6) with previously isolated compound (5) the minor signals typically as in compound (5) but the major peaks were agree with apigenin 7-*O*-β-D-glucopyranoside which show A2B2 spin coupling protons at δ_H_ 7.96 and 6.94 (d, *J* = 8.8 Hz) assigned for H-2′/6′) and (H-3′/5′) of B ring respectively also two meta doublet protons downfield ( ≈∆+0.2-0.4)ppm at δ_H_ ( 6.84 and 6.45 ) with *J* = 2.08 Hz assigned for H-8 , H-6 respectively on A –ring which confirm the glycone at C-7 , together with an olefinic protons at δ_H_ 6.97 singlet assigned for H-3 on a flavone C-ring in addition a signal at δ_H_ 5.07 (1,H,d, *J* =7 Hz) assigned for β glucopyranosyl anomeric proton(H"). compound (6) is predicted to be Apigenin 7-*O*-glucopyranoside. Confirmed by the downfield shift of H-8 and H-6 (≈∆+0.2-0.4) ppm and by the value of C-7 in ^13^CNMRspectrum.**^13^C NMR spectrum showed**: 13 carbon resonance were agreement with (Apigenin -7-*O*-β-D –glucopyranoside) [1] and [2] finally compound (6) were identified as minor compound (Acacetin 7-*O*-β-D –glucopyranoside) and major compound (Apigenin 7-*O*-β-D –glucopyranoside).

**Compound 7; ^1^H NMR** showed two doublets at 7.32 and 6.15 with large proton coupling *J* = 16.15 Hz were assigned to a pairs of trans olefinic protons H7and H8. In addition there were 2 ABX spin system observed in aromatic region ,which assigned to the two discrete sets of protons of the 3,4-dihydroxy phenyl unit, ortho coupled protons at 6.73 (1H*, d, J* = 8.5Hz) and 6.57 (1H, *d, J* = 8.5 Hz) assigned for H-5 and H-5׳ respectively and ortho, meta coupled protons at 6.93 (1H, *dd , J* =7.65 and1.7 Hz) and 6.47 (1H, *dd, J* = 7.65 Hz,1.7) assigned for H6 and H6׳ respectively, and finally meta coupled protons at 7.01 (1H, *d, J* =1.7 Hz) and 6.64 (1H, *br d*) assigned for H2 and H2׳ respectively. From previous and published data by and[4] Compound (7) identified as rosmarinic acid .

**Supplementary references**:

1. Markham, K. R. (1982). Techniques of flavonoid identification. Academic press.

2. Refaey, M. S., Abdelhamid, R. A., Elimam, H., Elshaier, Y. A. M. M., Ali, A. A., & Orabi, M. A. A. (2021). Bioactive constituents from Thunbergia erecta as potential anticholinesterase and anti-ageing agents: Experimental and in silico studies. *Bioorganic Chemistry*, *108*(January), 104643. https://doi.org/10.1016/j.bioorg.2021.104643

3. Lu, Y., & Foo, L. Y. (1999). Rosmarinic acid derivatives from Salvia officinalis. *Phytochemistry*, *51*(1), 91–94. https://doi.org/10.1016/S0031-9422(98)00730-4

4. Akoury, E. (2017). Isolation and Structural Elucidation of Rosmarinic Acid by Nuclear Magnetic Resonance Spectroscopy. *American Research Journal of Chemistry*, (June). https://doi.org/10.21694/2577-5898.17003
